# QTLs and Potential Candidate Genes for Heat Stress Tolerance Identified from the Mapping Populations Specifically Segregating for *F*_v_/*F*_m_ in Wheat

**DOI:** 10.3389/fpls.2017.01668

**Published:** 2017-09-27

**Authors:** Dew Kumari Sharma, Anna Maria Torp, Eva Rosenqvist, Carl-Otto Ottosen, Sven B. Andersen

**Affiliations:** ^1^Molecular Plant Breeding, Section for Plant and Soil Science, Department of Plant and Environmental Sciences, Faculty of Science, University of Copenhagen, Frederiksberg, Denmark; ^2^Section for Crop Sciences, Department of Plant and Environmental Sciences, Faculty of Science, University of Copenhagen, Taastrup, Denmark; ^3^Plant, Food & Climate, Department of Food Science, Aarhus University, Årslev, Denmark

**Keywords:** candidate genes, chlorophyll fluorescence, *F*_v_/*F*_m_, heat tolerance, phenotyping, photosynthesis, QTL, wheat

## Abstract

Despite the fact that *F*_v_/*F*_m_ (maximum quantum efficiency of photosystem II) is the most widely used parameter for a rapid non-destructive measure of stress detection in plants, there are barely any studies on the genetic understanding of this trait under heat stress. Our aim was to identify quantitative trait locus (QTL) and the potential candidate genes linked to *F*_v_/*F*_m_ for improved photosynthesis under heat stress in wheat (*Triticum aestivum* L.). Three bi-parental F_2_ mapping populations were generated by crossing three heat tolerant male parents (origin: Afghanistan and Pakistan) selected for high *F*_v_/*F*_m_ with a common heat susceptible female parent (origin: Germany) selected for lowest *F*_v_/*F*_m_ out of a pool of 1274 wheat cultivars of diverse geographic origin. Parents together with 140 F_2_ individuals in each population were phenotyped by *F*_v_/*F*_m_ under heat stress (40°C for 3 days) around anthesis. The *F*_v_/*F*_m_ decreased by 6.3% in the susceptible parent, 1–2.5% in the tolerant parents and intermediately 4–6% in the mapping populations indicating a clear segregation for the trait. The three populations were genotyped with 34,955 DArTseq and 27 simple sequence repeat markers, out of which ca. 1800 polymorphic markers mapped to 27 linkage groups covering all the 21 chromosomes with a total genome length of about 5000 cM. Inclusive composite interval mapping resulted in the identification of one significant and heat-stress driven QTL in each population on day 3 of the heat treatment, two of which were located on chromosome 3B and one on chromosome 1D. These QTLs explained about 13–35% of the phenotypic variation for *F*_v_/*F*_m_ with an additive effect of 0.002–0.003 with the positive allele for *F*_v_/*F*_m_ originating from the heat tolerant parents. Approximate physical localization of these three QTLs revealed the presence of 12 potential candidate genes having a direct role in photosynthesis and/or heat tolerance. Besides providing an insight into the genetic control of *F*_v_/*F*_m_ in the present study, the identified QTLs would be useful in breeding for heat tolerance in wheat.

## Introduction

Wheat (*Triticum aestivum* L.) being the third most important food crop in the world provides 20% of total calories and protein to the world population and its future productivity may influence global food security (Hawkesford et al., 2013). Sensitivity to heat stress is a major limitation to growth and productivity of wheat as especially in sub-tropical and dry regions, episodes of heat waves in combination with drought are serious during the anthesis and grain-filling period, which is the most vulnerable stage affecting the final yield (Ortiz et al., 2008). Even in most of Europe, heat stress during this sensitive developmental stage has been identified as a threat, thereby highlighting the importance of heat tolerance during anthesis as a key trait for improving yield potential and stability in wheat for future climate scenarios (Stratonovitch and Semenov, 2015).

Increasing the wheat productivity through selection for yield *per se* is slow because yield is a complex trait, highly affected by interaction between genotype and environment (Reynolds et al., 2012). In particular, transfer of heat tolerance from foreign to locally adapted wheat material is slow with conventional selection. An approach to identify and develop appropriate phenotyping techniques to measure heat tolerance related traits combined with improved understanding of the genetics of such traits would speed up the progress in breeding for stress tolerance. Genomic regions associated with heat tolerance have been previously mapped using quantitative trait locus (QTL) analysis for heat tolerance indicative traits such as a heat susceptibility index based on grain filling duration, thousand grain weight, yield and canopy temperature depression (Paliwal et al., 2012), stay green and senescence related traits (Vijayalakshmi et al., 2010; Kumari et al., 2013), thylakoid membrane damage, plasma membrane damage and chlorophyll content (Talukder et al., 2014) and grain weight stability associated with stay green (Shirdelmoghanloo et al., 2016).

In our studies, we have focused on unraveling the existing natural genetic variation in hexaploid wheat cultivars of diverse geographic origins for identifying potential QTLs/candidate genes for improving photosynthetic efficiency under heat stress by following a unique top-to-bottom three tiered physiological phenotyping combined with a quantitative genetic approach. In the first tier, phenotyping of a total of 1274 wheat cultivars belonging to different regions of the world was done repeatedly for three times under heat stress of increasing severity in order to identify the most consistently extreme performing cultivars solely based on the chlorophyll fluorescence parameter *F*_v_/*F*_m_, which indicates maximum quantum efficiency of photosystem II (PSII) photochemistry (Sharma et al., 2012). In the second tier, the identified cultivar differences for *F*_v_/*F*_m_ was validated for other physiological traits, showing that the cultivars selected for high *F*_v_/*F*_m_ were able to maintain higher overall net photosynthesis and dry matter accumulation under heat stress as compared to the low *F*_v_/*F*_m_ cultivars (Sharma et al., 2015). The third tier constituting the present study deals with the identification of genomic regions associated with the physiological differences through QTL mapping followed by identification of potential candidate genes. The uniqueness in the present study is that the three mapping populations used for QTL analysis have been derived from the three cultivars (origin: Afghanistan and Pakistan, therefore termed exotic in Denmark) selected as heat tolerant (maintaining high *F*_v_/*F*_m_) and one cultivar (origin: Germany) selected as the most heat sensitive (maintaining lowest *F*_v_/*F*_m_) under heat stress in our previous two tiers. Thus, the resulting F_2_ mapping populations were specifically segregating for *F*_v_/*F*_m_.

Chlorophyll *a* fluorescence techniques are widely used for rapid, non-invasive *in vivo* measurement of the physiological status of PSII under various environmental stresses (Maxwell and Johnson, 2000; Baker and Rosenqvist, 2004). The technique has been used to detect and quantify damage in PSII as a measure of heat tolerance in several crops including wheat (Sharma et al., 2012), tomato (Zhou et al., 2015), legumes (Herzog and Chai-Arree, 2012), barley (Rizza et al., 2011), and maize (Sinsawat et al., 2004). The QTLs associated with various chlorophyll fluorescence traits have been reported under drought stress (Zhang et al., 2010; Czyczylo-Mysza et al., 2011; Osipova et al., 2015) but rarely on heat stress (Azam et al., 2015), which was at seedling stage. The focus in the present study was heat stress (40°C) around anthesis, being a critically sensitive stage. The negative effect of heat stress in wheat is manifested by shortening of the grain filling duration, which is primarily because of the limited supply of photosynthetic assimilates either due to the reduced efficiency of heat damaged photosynthetic apparatus in itself and/or via loss of chlorophyll.

It is well documented that photosynthesis declines at temperatures well below the lethal level, although the underlying mechanism remains unclear. However, the photochemistry of PSII (in the light reaction) and the activation of Rubisco (in the dark reaction) are considered the most heat sensitive components of the photosynthetic apparatus (Heckathorn et al., 1997; Haldimann and Feller, 2004). Therefore, by using phenotyping by *F*_v_/*F*_m_, it was possible to identify the genotypic variations for the ability to withstand the heat stress damage (Sharma et al., 2012, 2014), which also reflected the overall photosynthesis and dry matter accumulation ability (Sharma et al., 2015). In spite of the genetic variation in photosynthetic efficiency in plants and the interaction of photosynthesis with the environment, genes responsible for the photosynthesis variation in the plant genetic resources are largely unexplored (Flood et al., 2011). Identification of molecular markers linked to such naturally existing sources of tolerance would facilitate breeding for photosynthetic efficiency during heat stress and thereby breeding for heat stress tolerance in wheat.

## Materials and Methods

### Plant Materials

Four parental lines were derived through three cycles of selection for *F*_v_/*F*_m_ during heat stress from originally 1274 spring wheat cultivars of diverse geographical origin (Sharma et al., 2012). Out of these four cultivars, three cultivars named 810 (IPK-2845, origin Afghanistan), 1039 (IPK-8183, origin Pakistan), and 1313 (IPK-28703, origin Pakistan) were selected as heat tolerant parents owing to their consistent high *F*_v_/*F*_m_ during all three heat stress screenings (Sharma et al., 2012) as well as in the subsequent physiological validation for maintaining higher overall photosynthesis (Sharma et al., 2015). Similarly, the fourth cultivar named 1110 (IPK-9705, Kloka WM1353, Germany) was selected as the most heat susceptible cultivar owing to its consistent low *F*_v_/*F*_m_ and net photosynthesis under heat stress in our previous studies (Sharma et al., 2012, 2015). Bi-parental crossings were made with each of these three heat tolerant cultivars (810, 1039, and 1313) as male parent and the heat susceptible cultivar (1110) as the common female parent. Seeds of a single F_1_ plant from each cross were then selfed to produce F_2_ progeny. A total of 140 F_2_ individuals in each of the three bi-parental cross combinations 1110 × 810, 1110 × 1039, and 1110 × 1313 constituted the three mapping populations.

### Phenotypic Evaluation of Mapping Populations under Heat Stress

A total of 420 F_2_ plants across three populations together with 10 plants each of the four parental cultivars (i.e., 460 plants in total) were completely randomized and divided into five blocks with 92 plants each. To be able to heat stress all the plants around anthesis seeds were sown 1 week apart between the blocks. Seeds were sown individually in plastic pots (11 cm diameter; 0.59 L) TEKU^®^, VCD series, Pöppelmann, GmbH & Co. KG, Kunststoffwerk, Werkzeugbau, Germany) with peat substrate (Pindstrup 2, Pindstrup Mosebrug A/S, Denmark) under greenhouse conditions at 15 ± 3°C day and 12°C night temperatures, 50–70% relative humidity and ambient CO_2_ concentration with regular irrigation and standard nutrient solutions consisting of 185 ppm (w/v) N, 27 ppm (w/v) P, 171 ppm (w/v) K, 20 ppm (w/v) Mg, and full micro nutrients, electric conductivity 2.0 mS m^-1^ and pH 5.8.

Phenotypic evaluation for heat tolerance was performed by following the previously developed protocol, which was standardized while screening the parental cultivars (Sharma et al., 2012). Plants in each block were transferred to a growth chamber (MB-teknik, Brøndby, Denmark) with 16 h of photoperiod at 250–300 μmol m^-2^ s^-1^ photosynthetic photon flux density (PPFD), 400 ppm CO_2_ and 60–70% RH. The intention behind the relatively low PPFD was to avoid the confounding effect of photo-inhibition on the heat stress treatment. Plants were acclimated at 20°C for 3 days before the temperature was increased 10°C per hour to 40°C (day/night) and kept constant for three consecutive days as heat stress treatment. The phenological stage of each plant was registered according to the BBCH-scale for cereals (Lancashire et al., 1991) and the heat stress treatment was given when the majority of the plants reached the growth stage in the range between heading and beginning of anthesis (BBCH scale 51–61) on day 0 of the treatment. The plants were watered frequently to avoid drought stress. During the acclimatization period, two penultimate leaves on each plant were fixed as the sampling leaves for measurements. Two measurements per plant were taken on day 0 (before transferring to growth chambers as control), day 1, day 2, and day 3 of heat stress. For each measurement, a leaf segment (5–8 cm long) was detached from each sampling leaf followed by clipping with a dark adaptation leaf clip (Hansatech Instrument, King’s Lynn, England). The clipped leaf samples were arranged on a plastic tray lined with moist tissue paper and enclosed in a plastic bag in order to avoid evaporative water loss during the 30 min of dark adaptation at room temperature (∼22°C). *F*_v_/*F*_m_ was measured on the adaxial leaf surface with a saturating flash of 3000 μmol m^-2^ s^-1^ for the duration of 1 s using a Plant Efficiency Analyzer, Handy PEA (Hansatech Instrument, King’s Lynn, England). The *F*_v_/*F*_m_ data were power transformed [(Y ^λ^ - 1)/λ] in order to obtain approximate uniform normal distribution of residuals across all the values (Box and Cox, 1964). The λ value was 11, which was initially determined from residual plots created using SAS ver. 9.2 (SAS Institute Inc., Cary, NC, United States). The two measurements from each day were averaged after the transformation and the data from the five blocks were pooled. For each of the parental lines (*n* = 10) and mapping populations (*n* = 140) the effect of heat stress over the duration of 3 days was calculated with paired *t*-test at *p* < 0.05. Since there were no replicates, the interaction between the block and genotype could not be tested. However, when the same growth chambers and experimental setups were used in our previous three-tiered phenotyping experiments to identify the parental lines no significant block effect was found in any of the three experiments and thus, the data could be pooled (Sharma et al., 2012).

### Isolation of Genomic DNA

Leaf samples from 1-month-old seedlings of each of the 460 plants used for phenotypic evaluation were freeze dried for 72 h using a freeze dryer (Christ Alpha 1-4, GmbH, Germany). The genomic DNA was isolated using the method described by Orabi et al. (2014) with slight modifications where, DNA was fished out using an inoculation loop followed by washing with 75% ethanol. Then pellets were air dried and suspended in 100 μl of TE buffer pH 8 (10 mM Tris.HCl and 1 mM EDTA) and allowed to dissolve at 4°C for a week. Concentration of the extracted DNA was measured with a nanodrop 2000 UV-Vis spectrophotometer (Thermo Fisher Scientific, Wilmington, United States) and the purity and integrity of the DNA was tested using 1% agarose gel electrophoresis.

### Genotyping of Mapping Populations

The DNA samples (50 ng μl^-1^ in TE buffer pH 8) from all the 460 plants were outsourced to the Diversity Arrays Technology Pty. Ltd. (DArT P/L, Yarralumla, ACT, Australia)^1^. The samples were genotyped using wheat DArTseq, a genotyping by sequencing platform that provides high-density single nucleotide polymorphism (SNP) as well as presence/absence variation (PAVs also called SilicoDArTs) markers. Additionally, this platform also provided the 69-nucleotide DNA sequence of the representative fragments derived from the genome complexity reduction process achieved through the use of a combination of restriction enzymes (Kilian et al., 2012). This high throughput genotyping on our mapping populations provided a total of 34955 DArTseq markers out of which, 30178 were PAVs and 4777 were SNPs.

### Linkage Map Construction and QTL Detection Based on Only SNP Markers: The First Round

SNPs scores were re-coded to reveal parental origin (“A” referring to the common female parent 1110) for each of the three populations for co-dominant (A, B, H) and dominant (either A/C or B/D) by a custom Java-based personal program (developed by SA). From the original 4777 SNPs, monomorphic as well as SNPs showing highly distorted segregation were eliminated. The linkage groups for the three mapping populations, 1110 × 810 (with 1042 SNPs), 1110 × 1039 (with 790 SNPs), and 1110 × 1313 (with 877 SNPs) were constructed using JoinMap^®^ 3 (Van-Ooijen and Voorrips, 2001). In the absence of information on the chromosomal location of these SNPs during this first round of analysis (in 2013), maps were constructed from linkage groups containing 2–100 markers at increasing log of odds (LOD) scores. A total of 39, 34, and 54 linkage groups were obtained in the 1110 × 810, 1110 × 1039, and 1110 × 1313 populations, respectively. These linkage maps were used for the QTL mapping of *F*_v_/*F*_m_ in order to check the presence of any significant QTLs using a Java-based software, SuperQTL (developed by SA). This software was based on multi-QTL interval mapping (Jansen and Stam, 1994) and was also used in a previous study (Kuzina et al., 2011). The LOD threshold for declaring a QTL significant was estimated using 1000 datasets with permuted trait values at *p* < 0.05 and was found to be 4.1 in the 1110 × 810, 4.4 in the 1110 × 1039, and 4.3 in the 1110 × 1313 populations (Supplementary Table S1).

### Final Linkage Map Construction Based on SNP, SSR, and PAV Markers

The sequence information of the important SNPs around the QTL region (from the first round) was used to retrieve the sequence of corresponding wheat contigs from the CerealsDB 2.0 database (Wilkinson et al., 2012). These sequences were subsequently mapped *in silico* to the bread wheat chromosome-based survey sequences in URGI^2^, to local databases of the wheat A (Ling et al., 2013) and D (Jia et al., 2013) draft genome sequences established in CLC main workbench^3^ (last date of access to the various databases: July 16, 2013). This bioinformatics approach provided us a hint that the identified QTL regions might be in the short arms of chromosome group 3, and group 1.

To confirm this finding, primer information for a total of 50 simple sequence repeats (SSRs) from the chromosome group 3 and 21 SSRs from the chromosome group 1 were searched from the database, Graingenes 2.0^4^ (Roder et al., 1998; Somers et al., 2004). Polymerase chain reaction (PCR) for SSR analysis was done with the three-primer approach for fluorescent labeling of the PCR products (Schuelke, 2000). PCR amplification was carried out as described by Orabi et al. (2014) and the SSR fragments were analyzed by capillary electrophoresis using a 3130xl Genetic Analyzer (AB/Hitachi, Thermo Fisher Scientific Inc., MA, United States). Out of a total of 71 SSRs tested, 12 SSRs from chromosome 3 and 15 SSRs from chromosome 1, were found polymorphic and hence, used for genotyping the three mapping populations.

In the meantime, a consensuses map on the genetic location of DArTseq markers (Li et al., 2015) and updates on the URGI wheat genome assembly (see text footnote 2) and Ensembl Plants wheat^5^ became available. The 69-nucleotide DNA sequence of the 34,955 DArTseq markers was used for BLAST search against a local depository of wheat sequences based on the *Triticum_aestivum*.IWGSC1+popseq.30 (downloaded from URGI and Ensembl Plants wheat, updated until August 2015). Only those markers that had a defined chromosomal position, 100% sequence identity and alignment, no gaps and mismatches, lowest e-value and highest bit scores were filtered in order to assign the chromosomal location. Further, the linkage group and position of these markers (when available) in the wheat DArTseq map (Li et al., 2015) was also assigned. This information was then used in differentiating linkage groups to assign chromosomes during map construction. The markers having missing scores of more than 11% and/or showing distorted segregation (*p* < 0.001) were removed before any further analysis. Since all the PAVs, some SNPs and a few SSRs were dominant markers, a linkage map was first calculated based on only the co-dominant SNPs using the regression mapping algorithm with Kosambi mapping function in JoinMap^®^ 4.1 (Van-Ooijen, 2006). The grouping information obtained from this co-dominant SNPs map in each population was then referred while grouping the rest of the markers during final mapping.

The final mapping of the linkage groups as well as the QTL analysis was done by QTL IciMapping software V4.0 (Meng et al., 2015). With the BIN function the redundant markers were removed by the missing rate of 11% that left the markers with fewest/no missing scores as representative marker of the bin. Before binning, there were a total of 4154, 4301, and 3819 polymorphic markers genotyped on 140 F_2_ individuals each of the 1110 × 810, 1110 × 1039, and 1110 × 1313 populations, respectively. The output from the BINNING was then used to create linkage groups by LOD threshold of 20 using the MAP functionality. Ordering of markers within the group was done by nnTwoOpt algorithm, followed by rippling step with SAD (sum of adjacent distances) with a window size of 10. Rippling fine-tunes the order of the markers within the linkage groups by permutation of the number of markers specified in the window size at a time.

The resulting final linkage maps of each population were then used to scan for QTLs following the bi-parental population BIP functionality in the same QTL IciMapping software v4.0, which is based on the inclusive composite interval mapping (ICIM). The QTL analysis was done for the trait *F*_v_/*F*_m_ separately for day 0 (control), day 1, day 2, and day 3 of heat stress. The set parameters were “deletion” for missing phenotypes, mapping steps at 1 cM and probability in stepwise regression (PIN 0.001) with ICIM-ADD mapping method and LOD threshold at 3.

### Identification of Physical Position and Potential Candidate Genes

After confirming that the chromosomal location of the QTLs was 3B and 1D, the physical position of the markers (when available) was searched from Ensembl Plants wheat and URGI databases. The QTL region between the flanking markers was estimated based on the one LOD drop off interval both on 3B and 1D chromosomes. The marker sequences were blasted against the Ensembl Plants wheat (see text footnote 5) to find the Traes numbers of the genes present around the QTL regions. The 3B region was updated through wheat_Jbrowse^6^ (URGI wheat_Jbrowse released September 14, 2015) hosted at URGI. These Traes numbers were searched in the UniProt in TrEMBL^7^ (release 2015_09 of September 16, 2015 of UniProtKB/TrEMBL) to obtain more information including protein domain, family, molecular and biological functions of the potential candidate genes (last assessed of various databases December 2015). However, only those genes with known function and/or related to stress and photosynthesis were counted as potential candidate genes.

The figures on phenotypic evaluation and linkage maps were drawn by Sigmaplot v11 (Systat Software, San Jose, CA, United States) and MapChart v2.3 (Voorrips, 2002), respectively.

## Results

### Phenotypic Performance of the Parents and Mapping Populations

**FIGURE 1:**
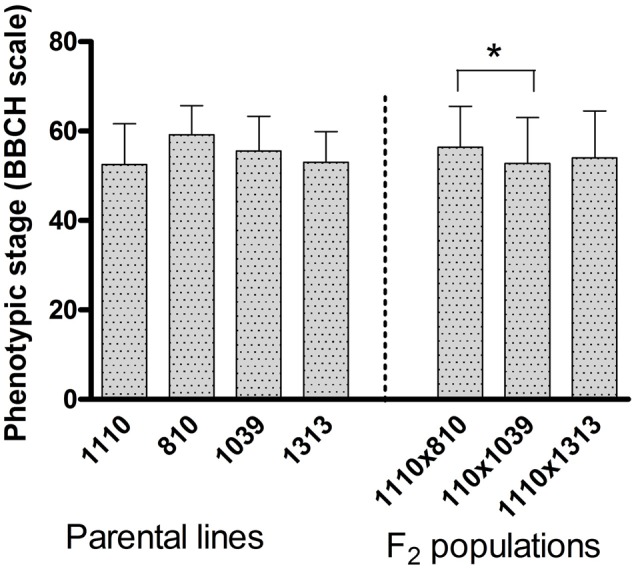
The average phenotypic growth stage according to the BBCH-scale for cereals registered on individual plants before subjecting them to heat stress (day 0). The error bar represents standard deviation (*n* = 10 for each parental cultivar and *n* = 140 for each mapping population). Significant difference between the four parental cultivars and three mapping populations is indicated by ^∗^ at *p* < 0.05.

The average phenological growth stage as per BBCH-scale for cereals (Lancashire et al., 1991) of the plants at the time of phenotypic evaluation (day 0) was considered (**Figure 1**). In the population 1110 × 810, the two parents respectively had a growth stage of 53 and 59, while the F_2_ population maintained stage 56, showing a non-significant variation between the two parents as well as between the F_2_ plants within the population. In the population 1110 × 1039, the heat tolerant parent 1039 and the F_2_ population were on growth stage of 56 and 53, respectively with a non-significant variation. In the third population 1110 × 1313, the growth stage was 53 in both the parents and 54 in the F_2_ population showing a non-significant variation (**Figure 1**). Overall, the growth stage ranged between 53 and 59 in the parental lines and 53 and 57 in the F_2_ populations (**Figure 1**), where the BBCH scale between 51 and 61 corresponds to the stage between inflorescence emergence (heading) and beginning of anthesis. All these non-significant variations demonstrate that the four parental cultivars were fairly uniform in their developmental rates and therefore, there was no clear segregation for phenological growth stage in their respective F_2_ population. However, comparison of the three populations shows that the 1110 × 810 population in average was slightly earlier than the 1110 × 1039 to reach anthesis (**Figure 1**).

**FIGURE 2:**
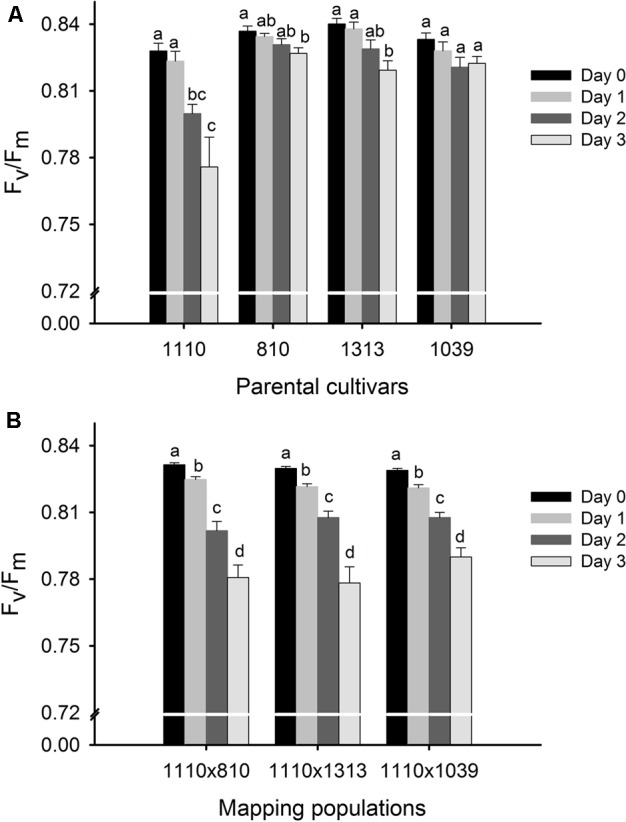
Phenotypic evaluation of four parental cultivars **(A)** and three F_2_ mapping populations 1110 × 810, 1110 × 1039, and 1110 × 1313 **(B)** before (day 0) and during 3 days of heat treatment at 40°C around anthesis using chlorophyll fluorescence trait, *F*_v_/*F*_m_ as a measure of heat tolerance. Four parental cultivars were previously selected for their consistently high (810, 1039, 1313—used as three male parents) and low (1110—common female parent) *F*_v_/*F*_m_ under heat stress and subsequently validated to be heat tolerant and heat susceptible parents, respectively. The effect of heat stress at *p* < 0.05 between the 3 days of treatment are indicated by different letters (*n* = 10 for each parental cultivar and *n* = 140 for each mapping population).

All the plants maintained a fairly constant *F*_v_/*F*_m_ around 0.84 at day 0 (20°C, control condition) while the 3 days heat stress treatment at 40°C led to a decrease in *F*_v_/*F*_m_ (**Figure 2A**). However, the heat stress induced decrease in *F*_v_/*F*_m_ was modest in the three tolerant parents, becoming significant only on day 3 in 810 (with 1.2% decrease) and 1313 (with 2.5% decrease) while being non-significant in 1039 (**Figure 2A**). On the other hand, there was a successive reduction in *F*_v_/*F*_m_ in the heat susceptible parent 1110 as the duration of heat stress progressed, with overall reduction of 6.3% on day 3 as compared to its day 0 value (**Figure 2A**). The three mapping populations showed an intermediate response, indicating a clear segregation for the trait, with the overall reduction ranging between 4 and 6% on day 3 as compared to their respective day 0 values (**Figure 2B**).

### Linkage Maps and Identified QTLs for *F*_v_/*F*_m_ and Potential Candidate Genes

Out of a total of 34955 DArTseq markers (including 4777 SNPs and 30178 PAVs) plus 27 polymorphic SSRs resulting from the genotyping of three mapping population, the final linkage map constructed using QTL IciMapping software V4.0 consisted of 1651 markers in 1110 × 810, 1752 markers in 1110 × 1039, and 1672 markers in 1110 × 1313 populations (**Table 1** and Supplementary Table S2). In all the populations, there were a total of 27 linkage groups covering all the 21 wheat chromosomes, confirmed by the information obtained from the genetic location of these markers in the wheat consensus map (Li et al., 2015) as well as from BLAST against the *Triticum_aestivum*.IWGSC1+popseq.30 genome assembly. However, in population 1110 × 1313 only two markers were mapped on chromosome 3D (**Table 1**). The chromosomes 3B, 6A, and 7A each formed two linkage groups. A small fraction (1–3%) of the total markers located to three short linkage groups with unknown chromosomal location termed not assigned (**Table 1**). The total length of genome across the three linkage maps was around 5000 cM with a marker distribution of 36–42% to the A genome, 47–51% to the B genome and 9–12% to the D genome.

**Table 1:** Distribution of markers on different chromosomes in the three populations. NA1, NA2, NA3—not assigned to chromosome.

		1110 × 810			1110 × 1039			1110 × 1313		
Linkage group	Chromosome	No. of marker	Length (cM)	Biggest gap (cM)	No. of marker	Length (cM)	Biggest gap (cM)	No. of marker	Length (cM)	Biggest gap (cM)
1	1A	90	308.37	20.33	122	289.28	40.17	142	214.22	16.71
2	1B	220	372.29	27.28	162	273.09	19.09	152	207.01	12.85
3	1D	37	238.06	73.00	41	361.11	64.71	65	336.77	66.66
4	2A	103	436.63	54.33	126	405.97	44.24	116	371.69	52.52
5	2B	134	312.37	55.72	195	231.56	18.08	180	293.65	15.19
6	2D	48	348.44	63.72	23	166.21	61.28	31	209.30	57.58
7	3A	126	278.76	16.23	113	212.60	27.72	100	412.97	33.46
8	3B	137	180.43	23.32	112	183.96	31.03	134	131.73	19.65
9	3B2	11	73.03	34.59	21	26.50	6.15	26	5.20	3.60
10	3D	19	72.96	34.12	15	204.17	65.81	2	0.00	0.00
11	4A	54	175.65	21.92	59	84.41	18.46	54	158.5	16.87
12	4B	55	223.12	69.97	26	143.62	39.32	20	152.55	42.26
13	4D	18	140.60	69.28	12	77.72	64.15	13	98.34	48.27
14	5A	29	155.59	23.08	60	180.82	42.86	55	186.13	31.75
15	5B	113	226.36	42.44	113	191.19	19.95	114	152.95	26.42
16	5D	17	166.92	35.65	28	171.52	50.68	18	126.38	65.85
17	6A	47	149.77	20.28	96	67.41	6.03	47	120.24	22.73
18	6A2	27	50.59	14.50	32	34.45	7.88	32	50.68	9.85
19	6B	88	132.77	10.74	66	120.22	29.6	64	155.93	28.94
20	6D	24	261.31	61.97	21	122.25	51.97	15	179.34	65.62
21	7A	94	263.65	61.48	97	215.01	37.51	100	352.21	31.38
22	7A2	24	50.96	12.40	28	77.88	7.50	44	47.81	6.58
23	7B	86	245.04	23.77	119	208.91	13.70	89	147.21	14.09
24	7D	28	147.87	48.90	15	115.76	54.95	13	173.14	52.89
25	NA1	7	10.26	4.75	14	4.58	2.32	11	23.80	6.72
26	NA2	10	8.53	2.51	17	18.14	2.58	12	16.45	5.99
27	NA3	5	16.73	13.57	19	21.86	12.20	23	56.51	11.59
A genome		594	1869.97	61.48	733	1567.83	44.24	690	1914.45	52.52
B genome		844	1765.41	69.97	814	1379.05	39.32	779	1246.23	42.26
D genome		191	1376.16	73.00	155	1218.74	65.81	157	1123.27	66.66
Not assigned		22	35.52	13.57	50	44.58	12.2	46	96.76	11.59
Total		1651	5047.06	73	1752	4210.20	65.81	1672	4380.71	66.66

**FIGURE 3:**
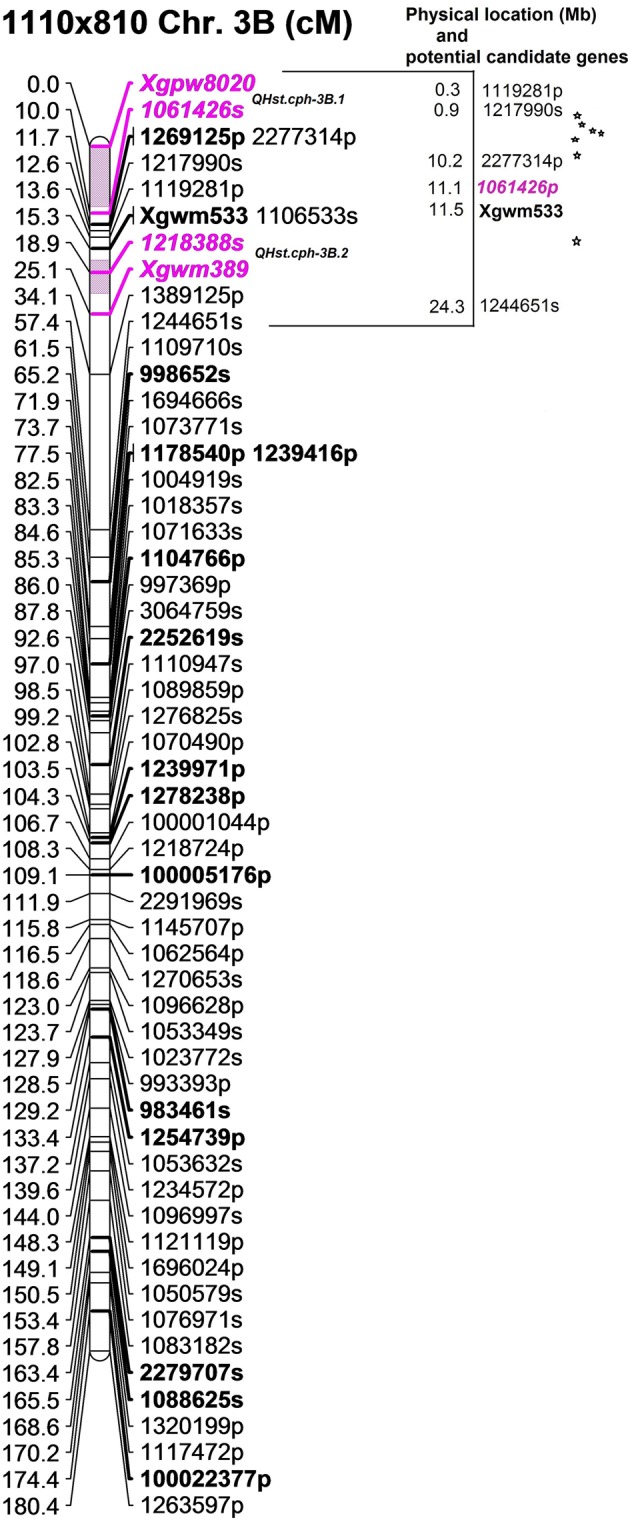
The position of the identified two QTLs (*QHst.cph-3B.1* and *QHst.cph-3B.2*) on chromosome 3B in the population 1110 × 810. The genetic map was created using 140 F_2_ genotypes. Only the markers with unique position (cM) are shown except at the flanking positions. Markers with a suffix “s” (denoting SNP) and “p” (denoting PAV) are DArTseq markers while markers with prefix “Xgwm” and “Xgpw” are SSR markers. The QTL region defined by one-LOD drop off interval is marked in color and the two flanking markers of the QTL peak are marked bold and Italics. Other bold markers indicate the common markers that were also mapped in the population 1110 × 1313. The corresponding approximate physical location (Mb) of the QTL region is indicated and each star within this region indicates a potential candidate gene having a known function.

QTL analysis using ICIM resulted in the identification of one significant QTL for *F*_v_/*F*_m_ in each population (**Table 2**). The QTLs in population 1110 × 810 (*QHst.cph-3B.1* and *QHst.cph-3B.2*) and 1110 × 1313 (*QHst.cph-3B.3*) were located on chromosome 3B while that in the population 1110 × 1039 (*QHst.cph-1D*) was located on chromosome 1D. For each of the three mapping populations the QTL region identified corresponded to the one found during the first round of mapping as shown by the presence of common flanking SNPs (Supplementary Table S2); with a slightly higher LOD score and percentage phenotypic variation explained but the same additive and dominance effects (**Table 2**). The LOD score of each of the three QTLs increased with increased duration of the heat treatment, making it significant only on day 3. There were no significant QTLs in other regions of the genome in any of the three populations (similar to the first round), except that the QTL peak on day 2 (*QHst.cph-3B.1*) shifted slightly upstream of the day 3 QTL (*QHst.cph-3B.2*) in the 1110 × 810 population (**Table 2**). In this population, the *QHst.cph-3B.2* peak was positioned at 20 cM flanked by markers *1218388s* (SNP) and *Xgwm389* (SSR), with the LOD score of 5.7 while the *QHst.cph-3B.1* peak with the LOD score of 6.4 was positioned at 5 cM flanked by markers *Xgpw8020* (SSR) and *1061426s* (SNP) (**Table 2**). The *QHst.cph-3B.1* and *QHst.cph-3B.2*, respectively explained about 22.1 and 25.4% of the phenotypic variation in the population with an additive effect of 0.002 and 0.003. The heat tolerant parent 810 donates the positive allele for *F*_v_/*F*_m_ in both the QTLs with the same dominance effect of 0.002 (**Table 2**). Using one-LOD drop off interval as an approximate confidence interval for the QTL position, the identified *QHst.cph-3B.1* and *QHst.cph-3B.2* QTLs spanned between 0 and 9 cM and 17 and 22 cM, respectively, where the two flanking markers were the only markers mapped at these particular intervals (**Figure 3**). However, there were more markers mapped in the vicinity (including the binned ones, a total of 28 markers mapped together at position 12 cM and six markers together at position 14 cM and eight markers together at position 15 cM on the current genetic map of 1110 × 810) (Supplementary Table S2). On the wheat physical map, the identified genomic region with the two QTLs corresponded to a region between 0.3 and 15 Mb (**Figure 3**). In this region, there were seven genes with known functions on the current assembly of wheat chromosome 3B hosted at URGI-Jbrowse database on traes3bPseudomoleculeV1 (**Figure 3**). These seven genes interestingly were all related to photosynthesis and heat stress and are therefore, considered as potential candidate genes identified from the *QHst.cph-3B.1* and *QHst.cph-3B.2* regions (**Table 3**). This includes *frk2* (fructokinase 2) and *bglu26* (beta-glucosidase 26) that are involved in carbohydrate metabolism. Further, there were two genes, *ndhB2* [chloroplastic NAD(P)H-quinone oxidoreductase subunit 2B] and *psaC* (photosystem I iron-sulfur center), both having a direct role in the photosynthetic light reaction (**Table 3**). Besides, there were a gene (*BUD31/G10* related) with a conserved site, and two genes encoding chloroplastic 3-isopropylmalate dehydrogenase 2) having known function in metal binding.

**Table 2:** Significant QTLs for *F*_v_/*F*_m_ during the 3 days of heat treatment at 40°C in the three F_2_ mapping populations.

Treatment	Population	Chromosome	Position (cM)	Left marker	Right marker	LOD	PVE%	Additive effect	Dominance effect	One-LOD drop off interval (cM)
Day 3	1110 × 810	3B	20	*1218388s*	*Xgwm389*	5.7	25.4	–0.003	0.002	17–22
	1110 × 1039	1D	49	*985618p*	*1698203p*	5.0	13.0	–0.002	0.0001	49–51
	1110 × 1313	3B	37	*1178540p*	*1127409s*	8.9	34.8	–0.003	0.001	35–39
Day 2	1110 × 810	3B	5	*Xgpw8020*	*1061426s*	6.4	22.1	–0.002	0.002	0–9

*Chromosomal position, flanking markers (left and right), maximum log of odds (LOD) score, percentage of phenotypic variation explained (PVE%), additive effect, dominance effect, and confidence interval as one-LOD drop off interval are provided. The negative values on additive effect indicate that the negative allele originates from the heat susceptible parent (1110) while the positive allele for F_v_/F_m_ is donated by the heat tolerant parents (810, 1039, and 1313) in all the identified QTLs*.

**Table 3:** List of 12 potential candidate genes having a known function (kf) related to photosynthesis and heat stress localized to the three identified QTL regions.

QTL name	Gene ID	Position (Mb) and direction	Length (bp)	Gene	TrEMBL Interpro description
*QHst.cph-3B.1* and *QHst.cph-3B.2*	*TRAES3BF060600270CFD_t1*	1.18+	1597	kf - *SCRK2_ORYSJ*	Fructokinase-2, *frk2*
	*TRAES3B100000020CFD_t1*	2.10+	2768	kf - *LEU32_ARATH*	3-Isopropylmalate dehydrogenase 2, chloroplastic
	*TRAES3BF093200180CFD_t1*	2.69-	2768	kf - *LEU32_ARATH*	3-Isopropylmalate dehydrogenase 2, chloroplastic
	*TRAES3BF093200170CFD_t1*	2.70+	2629	kf - *BGL26_ORYSJ*	Beta-glucosidase 26, *bglu26*
	*TRAES3BF093200040CFD_t1*	3.86+	2746	kf - *BD31A_ORYSJ*	*BUD31/G10*-related, conserved site (IPR018230)
	*TRAES3BF053100100CFD_t1*	9.45-	2186	kf - *NU2C2_LOLP*	Chloroplastic NAD(P)H-quinone oxidoreductase subunit 2B, *ndhB2*
	*TRAES3BF004500040CFD_t1*	14.56-	231	kf - *PSAC_VITVI*	Photosystem I iron-sulfur center, *psaC*
*QHst.cph-3B.3*	*TRAES3BF108400050CFD_t1*	30.56-	1896	kf - *PSB28_ORYSJ*	Photosystem II *Psb28*, class 1 (IPR005610)
*QHst.cph-1D*	*Traes_1DS_942B31C32*	14.47+	1229	*Peroxidase_WHEAT*	*Heme peroxidase* (IPR010255)
	*Traes_1DL_D5F3DA85C*	16.14+	1330	*αgalactosidase_WHEAT*	Glycoside hydrolase family 27
	*Traes_1DL_DF690B97B1*	17.39+	186	*PSBK_WHEAT*	Photosystem II *PsbK* (IPR003687)
	*Traes_1DL_776AF8007*	26.42-	7558	*DNAJ hsp_WHEAT*	DnaJ domain (IPR001623)

*Gene ID is the TRAES number according to the URGI-Jbrowse database on traes3bPseudomoleculeV1 for 3B and Ensembl Plants release 29—wheat chromosome 1D assembly for 1D (last assessed December 2015). +/- Sign indicates the direction on the strand.*

**FIGURE 4:**
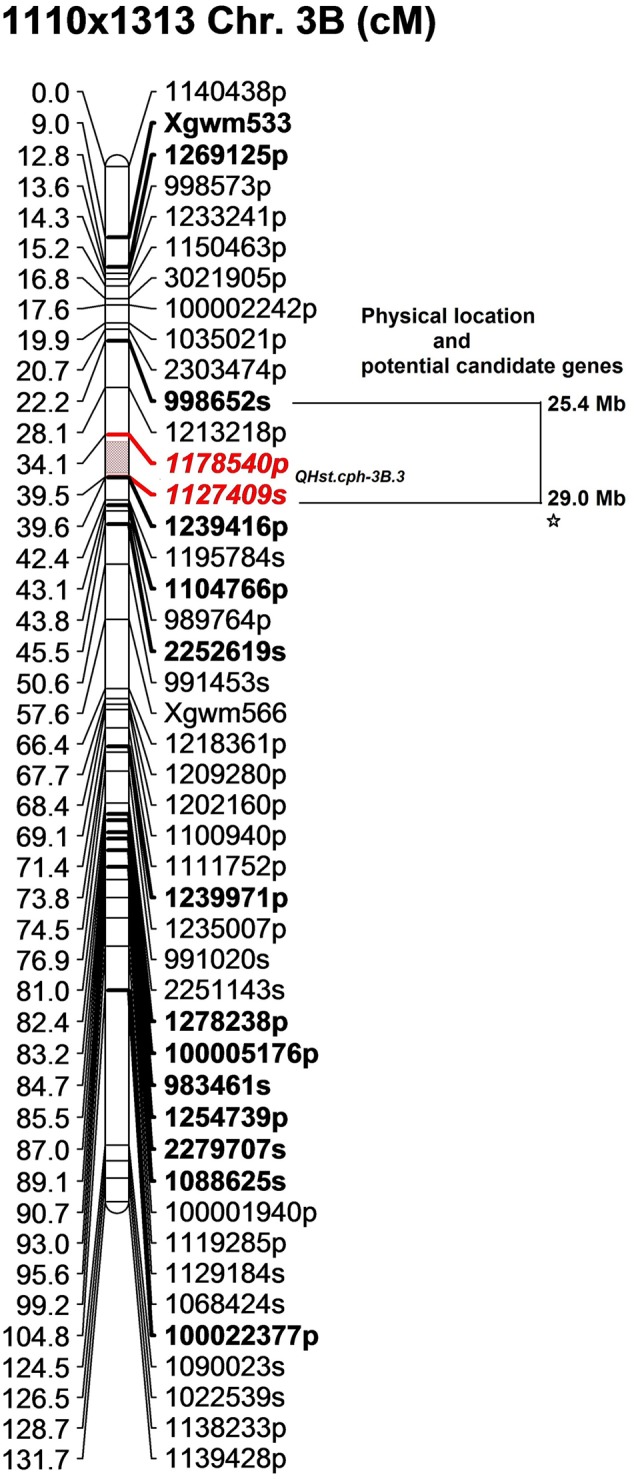
The position of the identified QTL (*QHst.cph-3B.3*) on chromosome 3B in the population 1110 × 1313. The genetic map was created using 140 F_2_ genotypes. Only the markers with unique position (cM) are shown (except at the QTL flanking region to show that *QHst.cph.3* is located slightly downstream of the *QHst.cph.1* and *QHst.cph.2*). Markers with a suffix “s” (denoting SNP) and “p” (denoting PAV) are DArTseq markers while markers with prefix “Xgwm” are SSR markers. The QTL region defined by one-LOD drop off interval is marked in color and the two flanking markers of the QTL peak are marked bold and Italics. Other bold markers indicate the common markers that were also mapped in the population 1110 × 810. The corresponding approximate physical location (Mb) of the QTL region is indicated and each star within this region indicates a potential candidate gene having a known function.

In the 1110 × 1313 population the identified QTL (*QHst.cph-3B.3*) for day 3 *F*_v_/*F*_m_ was located downstream of both *QHst.cph-3B.1* and *QHst.cph-3B.2* identified in the 1110 × 810 population on same chromosome, as indicated by the position of common markers mapped between the two mapping populations (compare bold markers in **Figures 3, 4**). This QTL was highly significant with a LOD score of 8.9 explaining 35% of the phenotypic variation for *F*_v_/*F*_m_ in the population. The heat tolerant parent 1313 contributed the positive allele for increasing *F*_v_/*F*_m_ in the population with an additive effect of 0.003 and dominance effect of 0.001 (**Table 2**). *QHst.cph-3B.3* was positioned at 37 cM flanked by markers *1175840p* (PAV) and *1127409s* (SNP) with one-LOD drop off interval between 35 and 39 cM on the 1110 × 1313 linkage map. This interval consisted of 11 markers including the binned ones. On the wheat physical map, this QTL region corresponded to a physical location approximately between 26 and 30 Mb, a region that holds a single potential candidate genes that have a known function and interestingly, it is *psb28*, class 1 that encodes a PSII reaction center protein (**Figure 4**). This protein is known to have a direct involvement in the oxygen evolving complex, biogenesis, assembly, stabilization, and repair of PSII complex (**Table 3**).

**FIGURE 5:**
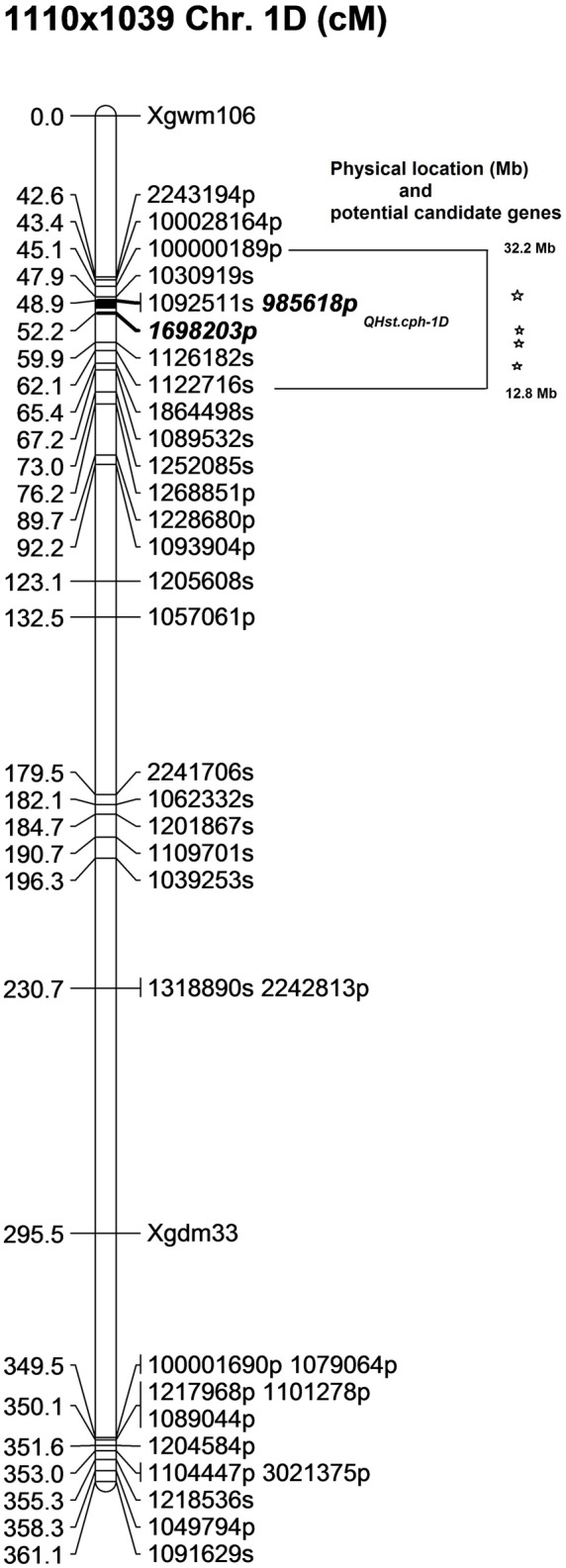
The position of the identified QTL (*QHst.cph-1D*) on chromosome 1D in the 1110 × 1039 population. The genetic map was created using 140 F_2_ genotypes. Markers with a suffix “s” (denoting SNP) and “p” (denoting PAV) are DArTseq markers while markers with prefix “Xgwm” and “Xgdm” are SSR markers. The QTL region defined by one-LOD drop off interval is marked and the two flanking markers of the QTL peak are marked bold and Italics. The corresponding approximate physical location (Mb) of the QTL region is indicated and each star within this region indicates a potential candidate gene having a known function related to photosynthesis and heat tolerance.

In the 1110 × 1039 population, the identified QTL (*QHst.cph-1D*) showed a LOD score of 5 and explained about 13% of the phenotypic variation for day 3 *F*_v_/*F*_m_ in the population. Similar to the other two populations, the source of heat tolerance originates from the tolerant parent 1039, contributing the positive additive effect of 0.002 and dominance effect of 0.001 (**Table 2**). The QTL peak is positioned at 49 cM on chromosome 1D with one-LOD drop off interval at 49–51 cM. The QTL is flanked by two PAV markers *985618p* and *1698203p* mapped at 48.9 and 52.2 cM, respectively (**Figure 5**). This interval including the binned markers holds four markers mapped in the 1110 × 1039 linkage map (Supplementary Table S2). The genetic location of *QHst.cph-1D* corresponded to an approximate physical location somewhere between 13 and 32 Mb on the assembly of wheat chromosome 1D hosted at Ensembl Plants (release 29, December 2015). This region holds four potential candidate genes considering only the genes having known functions related to photosynthesis and heat stress (**Figure 5**). These genes include *heme peroxidase* known to be involved in the response to oxidative stress; *αgalactosidase* that is involved in carbohydrate metabolism; *psbK* encoding a PSII reaction center protein having a direct involvement in the assembly, stability, and repair of PSII complex; a DNAJ hsp—a heat shock protein (**Table 3**).

## Discussion

In our approach, we have used a chlorophyll fluorescence trait, *F*_v_/*F*_m_, as a measure of heat tolerance. This trait reflects the maximum quantum efficiency of PSII to carry out photochemistry (Kitajima and Butler, 1975; Maxwell and Johnson, 2000; Baker and Rosenqvist, 2004) and this trait is linearly correlated with the maximum quantum yield of photosynthesis (Ogren and Sjöström, 1990). Therefore, any decrease in a fundamental process such as photochemistry may cause a negative effect, which would extend beyond PSII during the heat stress condition to cause a down regulation of overall photosynthesis and the total carbon gain of the plant. This was also evident in our previous study where, *F*_v_/*F*_m_ was positively correlated to both CO_2_ fixation and dry matter production (Sharma et al., 2015).

We have derived the mapping populations from three bi-parental crosses between the three consistent heat tolerant parents (810 and 1313, both originated from Pakistan, and 1039 originated from Afghanistan) and a common heat susceptible cultivar (1110, originated from Germany). These four parental cultivars were identified out of a pool of 1274 cultivars in our previous three-tiered screening under heat stress initially based on only *F*_v_/*F*_m_ (Sharma et al., 2012, 2014) followed by a subsequent validation for other physiological traits where it was found that the cultivars selected for high *F*_v_/*F*_m_ were also able to maintain high photosynthesis and dry matter accumulation under heat stress as compared to the cultivars selected for low *F*_v_/*F*_m_ (Sharma et al., 2015). There was a difference in photosynthesis rate between these heat tolerant and sensitive cultivars even at the same intracellular CO_2_ levels, indicating some intrinsic differences within the photosynthetic apparatus. The phenotypic evaluation of the parental and mapping populations also showed a differential reduction in *F*_v_/*F*_m_ during heat stress depending on their heat tolerance. Apparently, these differences in *F*_v_/*F*_m_ were not drastic in terms of value as a maximum difference of 9% between the tolerant and susceptible cultivars was seen in our various experiments. However, even a minor decrease in *F*_v_/*F*_m_ seems to reflect the genetic difference of a cultivar with respect to PSII functionality and the net photosynthesis rate during heat stress. This makes sense because such genetic factors associated with stress tolerance of a fundamental process such as photosynthesis are supposed to be conserved over the course of evolution, since types with reduced performance will be selected against. For this reason, it is not surprising that the genotypic differences for performance of such key physiological processes are rather small among cultivated wheat. However, the thorough three-tiered combined phenotyping and genetic approach allowed us to genetically localize QTLs for this trait and identify some potential candidate genes related to heat stress tolerance for a fundamental physiological process.

Two significant genomic regions on chromosome 3B and one on chromosome 1D associated with *F*_v_/*F*_m_ in the three mapping populations explained between 13 and 35% of the phenotypic variation signifying that *F*_v_/*F*_m_ is a genetically governed quantitative trait that reflects the cultivars’ ability to withstand heat stress at the level of PSII and photosynthesis. Remarkably, this genetic effect on the trait was heat stress driven since the QTL peak and its effects progressed with increasing duration of heat stress, with no QTL at day 0 control (20°C). This corroborates our previous assumption based on the estimates of genetic determination, where it was found that the genetic differences among cultivars’ *F*_v_/*F*_m_ arose only when heat stressed (Sharma et al., 2012). Interestingly, all the three heat tolerant male parents contribute the allele that increases *F*_v_/*F*_m_ during heat stress. This also means that the source of these identified QTLs is exotic for Scandinavian/European bread wheat owing to the origin of these male parents being Afghanistan and Pakistan. Possibly a natural selection may have been operating to conserve tolerant alleles in order to adapt these cultivars to the warmer growth conditions in their original habitat. The additive effect of the individual QTLs is small by value (0.002–0.003) but considering that the maximum possible value of *F*_v_/*F*_m_ is 0.85 (Maxwell and Johnson, 2000) and the estimated genetic determination of *F*_v_/*F*_m_ in the parental lines by the third round of selection was only 28% implying a low heritability (Sharma et al., 2012), it is not surprising that the additive effect of the identified QTLs are also small. However, even a minor reduction in *F*_v_/*F*_m_ is enough to create a significant difference in the net photosynthesis during heat stress (Sharma et al., 2015). It was found that even though the top 5 and bottom 5 cultivars selected out of a pool of 1274 cultivars differed in *F*_v_/*F*_m_ by only 9%, these cultivars showed a significant difference in net photosynthesis at light saturation by 20% in the subsequent experiments. Nevertheless, pyramiding of the identified QTLs might be a way forward to utilize these naturally existing genetic variations to improve photosynthetic efficiency under heat stress.

There have been some studies on mapping QTLs for chlorophyll fluorescence traits but most of these studies are done under drought stress (Zhang et al., 2010; Czyczylo-Mysza et al., 2011; Osipova et al., 2015) and rarely on heat stress (Azam et al., 2015). In drought stressed wheat plants, the co-localization of QTLs for number of grains and grain dry weight per main stem ear, and *F*_v_/*F*_m_ was found on chromosome 5A (Czyczylo-Mysza et al., 2011) while another study also found a QTL for *F*_v_/*F*_m_ on 3B under drought stress at the grain filling stage (Yang et al., 2007) This genomic region was also associated with *F*_m_ and *F*_v_ under heat stress occurring at seedling stage (Azam et al., 2015). Under heat stress various QTLs have been identified for other traits such as heat susceptibility index based on thousand grain weight, grain filling duration, canopy temperature depression at the terminal heat stress conditions in wheat and have found significant genomic regions on 2B, 7B, and 7D out of which two QTL (2B and 7B) jointly explained more than 15% of phenotypic variation (Paliwal et al., 2012). Similarly, nine QTLs were found on different chromosomes including 2A, 3A, 6A, 7A, 3B, and 6B for senescence related traits during post-anthesis heat stress in winter wheat (Vijayalakshmi et al., 2010).

In the present study, the *QHst.cph-3B.1* and *QHst.cph-3B.2* identified in the population 1110 × 810 appears to be separate QTLs while considering the peak position for day 2 (0–9 cM at the tip, where the two flanking markers are the only markers mapped in that interval) and day 3 (17–22 cM) slightly below. However, the physical position of some of the surrounding markers seemed to overlap (**Figure 3**), suggesting that they are possibly the same QTL. The genetic order and the physical order of the markers did not completely correspond in this particular region (**Figure 3**), which could be partly because the majority of the markers mapped in this region are dominant PAV markers. However, it is very interesting to mention that in this genetic region near the SSR marker *Xgwm533* (see **Figure 3**), six different studies have previously mapped QTL for traits such as staying green, chlorophyll content and prolonging grain filling duration under heat stress (as neatly compared by Shirdelmoghanloo et al., 2016). The *QHst.cph-3B.3* identified in the population 1110 × 1313, albeit on the same chromosome, is a separate QTL and is located downstream of *QHst.cph-3B.2*, and it corresponds to a region that holds *psb28* that encodes a PSII reaction center protein. Taken together, the results strongly suggest that the identified QTLs are potentially important for improving photosynthesis under heat stress.

In the present study, some of the linkage groups were apparently long with a large gap between some of the markers, thus giving a total length of genome of around 5000 cM with a marker distribution of 36–42% to the A genome, 47–51% to the B genome, and 9–12% to the D genome. However, a similar situation of having some of the linkage groups mapped long, presence of more than one linkage groups in some of the chromosomes and a less representation of the D genome have also been seen in other studies with DArTseq markers (Li et al., 2015). The fact that the present study dealt with F_2_ populations and a large number of dominant markers (particularly, the PAVs) is probably part of the explanation behind the long linkage groups, although the maps based on only the codominant markers were used as anchor/reference points during mapping to take this in to account. In addition, efforts were made to sort out the most problematic markers before mapping and deleted all markers with more than 11% missing values and highly distorted segregation (*p* < 0.001). Particularly for the linkage groups harboring QTLs (3B and 1D) several rounds of mapping including rippling of markers have been carried out to improve the maps and compare them to other available maps including the physical map of 3B.

The identified QTL regions in all the three populations is still large, particularly in term of approximate physical location, which is partly because of the unknown physical position for many of the markers. Nevertheless, amidst numerous genes having unknown functions, putative functions and hypothetical/uncharacterized proteins (Cunningham et al., 2015), the fact that identified QTLs also holds some of the genes directly associated with photosynthesis looks promising. In particular, *ndhB2* and *psaC* near the *QHst.cph-3B.1* and *QHst.cph-3B.2*; PSII reaction center protein coding genes *psb28* near *QHst.cph-3B.3* and *psbK* near *QHst.cph-1D* are highly relevant. They could potentially have a direct impact on maintaining high *F*_v_/*F*_m_ during stress because these proteins are known to be involved in the oxygen evolving complex, biogenesis, assembly, stabilization, and repair of PSII complex (Bateman et al., 2015). As disruption of the oxygen evolving complex is one of the effects of heat stress that leads to low photochemistry (Mathur et al., 2011), it is also likely that these genes helps the tolerant lines to maintain higher *F*_v_/*F*_m_ by buffering damage on the oxygen evolving complex. Furthermore, *frk2* and *bglu26* that are involved in carbohydrate metabolism as well as *heme peroxidase* involved in response to oxidative stress and a heat shock protein DNAJ (Bateman et al., 2015) are also potentially important. For a quantitative trait such as heat tolerance, it might be possible that many of these genes function in ordinance to maintain an efficient photosynthetic machinery to overcome heat stress damage. Therefore, it is possible that the cluster region identified on 3B and 1D could be one of the hot spots for photosynthesis related traits in hexaploid wheat.

As mentioned before, the sources (810, 1039, and 1313) of the identified QTL/genes being from Afghanistan and Pakistan, they are exotic to the Scandinavian region, so the parental cultivars as such are not expected to be adapted this Northern European climate. Thus additional efforts are needed to transfer the identified QTLs into the locally adapted cultivars through marker assisted backcrossing with the help of the identified flanking markers. Further work in the direction of fine mapping using recombinant inbred line populations where many recombinant events as well as replicated phenotypic evaluation might give better resolution of the QTL region as compared to the present study which was based on F_2_ populations. In addition, the on-going advancements in the wheat genome databases would aid in a better physical mapping of the markers (SNPs, PAVs, and SSRs) in the future thereby enabling more narrow localization of the QTL regions and pinpoint the exact candidate genes for the trait, which was still difficult with uncertain positions of many of the markers particularly for the 1D chromosome.

By a combined approach of three-tiered phenotyping using *F*_v_/*F*_m_ to select the four parental lines followed by a linkage analysis of SNP, PAV, and SSR markers in the three mapping populations specifically segregating for *F*_v_/*F*_m_, we have identified two important genomic regions on chromosome 3B and one on 1D associated with *F*_v_/*F*_m_ under heat stress around anthesis. Overall, these genes reveal differences in tolerance in the functionality of PSII under heat stress condition. These QTLs may be either further fine mapped to identify the exact candidate genes for the trait or the identified potential candidate genes as discussed above may be further investigated/targeted to improve the PSII efficiency during heat stress. This may improve our genetic understanding of heat tolerance in wheat through enhanced photosynthetic performance, while the QTL segments may be pyramided and directly used for breeding of more heat tolerant wheat cultivars through marker-assisted selection with the use of the identified flanking markers.

## Author Contributions

All the authors were involved in the planning and execution of the work. DS majorly conducted all the experiments, lab works, analyzed data in final round, and prepared the manuscript draft. SA helped DS in the generation of mapping populations and lab works, and majorly analyzed the genotypic data and QTL mapping in the first round and corrected the first draft of the manuscript. AT contributed in the bioinformatics works, helped DS in the final round of genotypic data analysis and corrected the manuscript. ER and C-OO helped DS in the phenotypic evaluation and corrected the manuscript.

## Conflict of Interest Statement

The authors declare that the research was conducted in the absence of any commercial or financial relationships that could be construed as a potential conflict of interest.

## Footnotes

1 http://www.diversityarrays.com

2 http://wheat-urgi.versailles.inra.fr/Seq-Repository

3 http://www.clcbio.com

4 http://wheat.pw.usda.gov/GG2/index.shtml

5 http://plants.ensembl.org/Triticum_aestivum/Info/Index

6 http://wheat-urgi.versailles.inra.fr/About-us/News/Use-JBrowse-to-display-the-3B-reference-sequence

7 http://www.ebi.ac.uk/uniprot
