# Supplementary material for: QTLs and Potential Candidate Genes for Heat Stress Tolerance Identified from the Mapping Populations Specifically Segregating for Fv/Fm in Wheat
Source: Front Plant Sci. 2017 Sep 27;8:1668. doi: 10.3389/fpls.2017.01668 (PMC5623722; doi:10.3389/fpls.2017.01668)
Supplement: Supplementary file 1 [file Table_1.DOCX]

| **Population** | **Treatment** | **LOD** | **PVE** | **Add. Effect (x10-4)** | **Dom. Effect (x10-4)** | **Peak (cM)** | **Flanking markers** | **CI (cM)** |
| --- | --- | --- | --- | --- | --- | --- | --- | --- |
| 1110x810 | Day 0 | 3.3 | 10 | -1.9 ± 2.5 | 9.9 ± 2.4 | 126 ± 6.5 | *1061426s*  *1218388s* | 16 |
|  | Day 1 | 1.7 | 5 | -1.2 ± 5.2 | 10.8 ± 6.1 |  |  |  |
|  | Day 2 | 5.1* | 15 | -15.4 ± 4.8 | 13.9 ± 6.6 |  |  |  |
|  | Day 3 | 5.6* | 16 | -19.5 ± 5.3 | 13.0 ± 9.6 |  |  |  |
| 1110x1313 | Day 0 | 2.6 | 8 | -2.3 ± 2.2 | 9.1 ± 6.8 | 86 ± 13.5 | *1127409s*  *998652s* | 81 |
|  | Day 1 | 2.1 | 7 | -5.1 ± 3.6 | 8.0 ± 12.4 |  |  |  |
|  | Day 2 | 3.7 | 11 | -19.1 ± 5.9 | -0.7 ± 9.2 |  |  |  |
|  | Day 3 | 4.2* | 12 | -15.4 ± 3.7 | -4.2 ± 11.9 |  |  |  |
| 1110x1039 | Day 0 | 0.9 | 3 | -2.8 ± 3.0 | -3.7 ± 9.0 | 52 ± 19 | *1864498s*  *1092511s* | 55 |
|  | Day 1 | 1.4 | 4 | -4.3 ± 3.6 | 9.8 ± 5.7 |  |  |  |
|  | Day 2 | 3.6 | 11 | -13.1 ± 3.3 | 7.6 ± 5.4 |  |  |  |
|  | Day 3 | 4.8* | 14 | -17.0 ± 3.8 | -0.1 ± 9.2 |  |  |  |

**Table S1:** Maximum log of odds (LOD) score, percentage phenotypic variation explained (PVE), additive (add.) effect, dominance (dom.) effect, QTL peak position, two flanking markers and confidence interval (CI) of the three identified QTLs for F_v_/F_m_ (in the first round of QTL analysis) before (Day 0) and during the three days of heat treatment. Significant LOD score at p<0.05 is indicated as ‘*’. The negative values on additive or dominance effects indicate that the negative allele originates from the heat susceptible parent (1110) while the tolerant parents (810, 1039 and 1313) in all the three mapping populations donate the favourable allele for F_v_/F_m_. ± value indicates standard error.
